# Jew and Gentile

**Published:** 1873-08

**Authors:** 


### JEW AND GENTILE.

ISRAELITES live and flourish in all countries and in all climes, beyond any other race and nation; the reason is, the God of their fathers promised it, even before they became a nation. At the same time the Ruler of the world uses instrumentalities, and accomplishes his designs by the operation of natural laws. A very large number of the observances imposed through Moses and Aaron had two main ends in view—to prove their obedience and to preserve their lives, to enable them to live healthily, happily and long. The result is, that of all the peoples who have ever lived the Jew only retains his name and nation and lineage. Hence their existence to-day is the greatest miracle connected with the Christian religion, for it is founded on the Bible, and the simple existence of the Jews as a people demonstrates the fact that the Bible is the Word of God.

Dr. Nenfville, of Frankfort—the birthplace of the Rothschilds—states that, in round numbers, the average duration of the life of the Israelites in that city is forty-nine years; that of the rest of the population is thirty-seven years.

During the first five years of life Jewish deaths are but little over one-half as many as Christian.

One fourth of Christian children die before they are seven years old, while three fourths of the Jews live to the age of twenty-eight years.

Only one quarter of the Christians live to be sixty years old ; a fourth of the Jews live to the age of seventy-one.

It is stated, on the authority of Glotter, that in the Austrian dominions, while of one thousand of other nationalities who live from seventy to a hundred years, there were seventy Croats and seventy-five Germans, while out of a thousand Jews, one hundred and twenty lived to over seventy years. The reasons usually assigned for these favorable results are, that the mode of life of the Jews, in reference to their eating, and drinking, and personal habits, tend directly and efficiently to this better showing.

There is, however, another element—that of general thrift. The ability to make money and save it, and have it on hand ready to make profitable investments, gives a certain degree of independence and quiet of mind, and a feeling of elevation, tending to the promotion of a pleasurable serenity that is worth millions of money.

It is for the same reason that the Friends, called Quakers, have an average of eleven years more than the "world's people." They are thrifty, clean, industrious, economical, and of a quiet, calm temperament. But there is to the Jew a sentiment, but only a sentiment, which gives to him a fixedness, a settled feeling, which is of great value—his unalterable faith that there

IS A GOD.

No people on the globe are farther from idolatry than are the Israelites ; their faith is fixed. No doubts, no uneasiness, no feeling about for the truth, for it is within them ; upon it they are willing to stake their existence in all the emergencies of life. It is emphatically true of them, "Their faith is fixed, trusting in Jehovah."

It then follows that the great promoters of longevity are :

First. Cleanliness.

Second. A proper mode of diet, consisting mainly in the avoidance of gross and fat foods.

Third. Pecuniary thrift.

Fourth. A fixed religious sentiment—a sentiment which, amid the perturbations and storm of life, enables its fortunate possessor to maintain a composed, and equable, and confident frame of mind.

But, as a people, the American—the representative Yankee—has no composure, no quietude, no serenity ; he has no rest until he finds it in the grave.

